# Coping, connection appraisal, and well-being during COVID-19 in the U.S., Japan, and Mexico

**DOI:** 10.3389/fpsyg.2024.1420327

**Published:** 2024-08-30

**Authors:** Laurel R. Benjamin, Shu-wen Wang

**Affiliations:** ^1^Department of Psychology, San Diego State University/University of California San Diego Joint Doctoral Program in Clinical Psychology, San Diego, CA, United States; ^2^Department of Psychology, Haverford College, Haverford, PA, United States

**Keywords:** COVID-19, mixed-method, coping, appraisal, well-being

## Abstract

**Introduction:**

The COVID-19 pandemic has affected nearly every facet of life, constituting a “new normal” and prompting an ongoing collective psychological crisis. People’s ways of coping with the pandemic and corresponding well-being are of particular research interest; however, these constructs have largely been examined using deductive quantitative approaches, deficit-based lenses, and mononational samples.

**Methods:**

The current mixed-methods study used inductive-sequential (QUAL → QUAN) approaches to explore positive coping strategies (approach coping style and COVID-related connection appraisal) and well-being (loneliness, distress, and happiness) across individuals from the United States, Japan, and Mexico. Qualitative data were gathered from *N* = 141 U.S., Japanese, and Mexican adults to examine how people perceived connection during the pandemic.

**Results:**

Qualitative analyses illuminated common themes in which people appraised the pandemic as an opportunity for connection and strengthened interpersonal relationships. Quantitative measures, including a newly-developed questionnaire on COVID-related connection appraisal, were then administered to a separate sample of *N* = 302 adults in the U.S, Japan, and Mexico to assess associations among approach coping style, COVID-related connection appraisal, and well-being outcomes (loneliness, distress, happiness). Quantitative analyses found significant associations among approach coping style, COVID-related connection appraisal, and all well-being outcomes. Of note, these associations did not differ by country. COVID-related connection appraisal mediated the relationship between approach coping style and two well-being outcomes (loneliness and happiness).

**Discussion:**

Findings point to approach coping style and connection appraisal as pathways for resilience and growth in the face of global suffering.

## 1 Introduction

We are currently in the midst of a communal and scientific reflective process over the unprecedented and ongoing impacts of the COVID-19 pandemic. Since March 11, 2020, when the World Health Organization (WHO) declared COVID-19 a global pandemic, nearly every facet of life all over the world has been impacted by this public health crisis. Not only has there been profound loss of human life (estimated to be 14.83 million excess deaths by the WHO; Msemburi et al., 2023), COVID-19 also precipitated waves of medical and economic crisis (e.g., Anoushiravani et al., 2023); has transformed social, interpersonal, and family processes (e.g., Benjamin and Wang, 2022; Cassinat et al., 2021; Williamson, 2020); and has triggered an ongoing collective psychological crisis (e.g., termed a “collective trauma”; Silver et al., 2021) that social scientists are still trying to understand.

The progression of COVID-19 research has also moved from earlier models based on previous global infections [e.g., Severe Acute Respiratory Syndrome (SARS) and Middle East Respiratory Syndrome (MERS); Serafini et al., 2020] and use of prior psychological theories to predict COVID-19 outcomes and processes (e.g., Abrams et al., 2021; Pietromonaco and Overall, 2021), to empirical research involving collection of COVID-19 era data to test questions and predictions. We now have more data and a fuller (retrospective) context in which to ask more nuanced questions about the substantial continuing impacts of COVID-19 on psychosocial functioning and individual well-being. Since the pandemic’s onset, a number of studies have been conducted to investigate the impact of the pandemic on personal well-being (e.g., loneliness, distress, happiness) and associated forms of coping across a wide range of clinical and community samples. Indeed, several cross-cultural studies have been conducted to examine the comparative psychosocial effects of the pandemic between countries, including the U.S., Japan, Malaysia, and China (Sugawara et al., 2022), Germany and Portugal (Candeias et al., 2021), and several Spanish-speaking countries (e.g., Spain, Mexico, Chile; Schoeps et al., 2023). Nonetheless, the bulk of existing COVID-19 research remains reliant on deductive and strictly quantitative approaches, predominantly examines the pandemic through a deficit-based lens, and largely investigates the effects of coping on well-being *within* a single given country or cultural context, thus calling into question the external validity and universality of these findings. The current study, therefore, seeks to address these gaps by examining well-being and coping during COVID-19 using mixed-method, multi-national, and positive psychological approaches.

When the pandemic first emerged, many governments throughout the world instituted a series of lockdowns and stay-at-home orders, urging citizens to remain at home and restrict their in-person social interactions to counter viral spread. Not only was this social isolation (or social distancing) positively associated with psychological distress, as has been found in a global sample of >13,000 older adults drawn from 62 countries during COVID-19 (Kim and Jung, 2021), but research evidence also indicates that this period of time saw overall negative impacts on relationship processes. For example, families have reported more “chaos” and more negative parenting strategies (Cassinat et al., 2021), and lower-functioning couples also displayed decreased relationship satisfaction and increased maladaptive attributions (Williamson, 2020). Overall, studies suggest that the challenges of living through the COVID-19 pandemic are linked with adverse mental health effects, including a 43% increase in symptoms of post-traumatic stress disorder, a 28% increase in anxiety, and a 27% increase in depression (Cao et al., 2022); in addition to 0.11-point decreases in life satisfaction and 0.09-point decreases in positive affect on standardized measures of personal well-being (Zacher and Rudolph, 2021).

Individual responses to the COVID-19 pandemic are shaped by multiple factors. According to Folkman and Lazarus (1988) transactional theory of coping, an individual’s psychological response to a stressful event is shaped by the individual’s cognitive appraisals of the stressor, which include both primary appraisals of the event (e.g., viewing the event as a threat, challenge, or loss) and secondary appraisals of one’s own ability to cope; these appraisals then influence emotional responses and subsequent behavior. Scholars have conceptualized common coping strategies in terms of the direction of the coping response in relation to the stressor, with *approach coping* encompassing cognitive and emotional activity that is oriented *toward* the stressor (i.e., problem solving, support seeking, positive reframing, planning, and acceptance), and *avoidant coping* referring to an orientation *away* from a stressor (i.e., denial, withdrawal, substance use, self-distraction, and self-blame; Roth and Cohen, 1986). Studies have indicated that avoidant coping (versus approach coping) is linked with poorer physical health outcomes and is less effective at attenuating anxiety symptoms (Eisenberg et al., 2012; Roth and Cohen, 1986), whereas approach coping is generally associated with better health outcomes, greater emotional adjustment and goal attainment, and may more effectively buffer against anxiety symptoms in response to a prolonged stressor (Eisenberg et al., 2012; Monzani et al., 2015; Roth and Cohen, 1986). As such, we contend that approach coping style, in particular, may serve as a potential pathway for resilience in the face of a prolonged global stressor like the COVID-19 pandemic.

An emerging body of literature has started to examine the association of approach coping style with well-being in the context of COVID-19, with findings indicating that greater trait levels of approach coping help to buffer against distress. One study found approach coping style to mitigate the distress of COVID-19 isolation in a sample of 1,749 youth from the United States and Australia (Cheng et al., 2021). Another similar study found that approach coping style was significantly related to lower depression and better quality of life among adults and found positive reframing to be the most beneficial form of approach coping during this time (Shamblaw et al., 2021). A related body of literature has also examined *interpersonal coping strategies* during COVID-19 as another contributor of well-being and even growth. Indeed, one study conducted among healthcare professionals in Hong Kong found that nurses with greater levels of interpersonal coping (e.g., perceiving oneself as having greater social support) experienced greater “adversarial growth” (i.e., positive changes resulting from stressful life events which surpass the pre-event level of functioning; Yeung et al., 2023). Another study found similar evidence of relational improvements in couples following the pandemic’s onset as a function of perceived interpersonal closeness and positive relational functioning (Vowels et al., 2021). Additionally, a study conducted among 544 adults during the COVID-19 lockdown period in Italy found that having appraisals oriented toward growth and supportive openness toward others contributed to improved self-efficacy and, in turn, lower levels of perceived distress (Diotaiuti et al., 2023). Both forms of coping (both more *trait-like* approach coping styles as well as more *COVID-specific* interpersonal coping) may be of particular benefit to individuals during the pandemic as sources of resilience and even growth. No published study, to our knowledge, has examined these two forms of coping concurrently and, hence, their comparative effects on well-being remain unknown. As such, the current study seeks to explore both forms of coping and to examine whether COVID-specific coping styles differ from traditional approach coping styles in their associations with well-being.

To date, pandemic coping and well-being outcomes have largely been examined using quantitative approaches with most researchers relying on existing scales and constructs to approximate the unprecedented experience of living during COVID-19. Mixed-method approaches, which incorporate qualitative inquiry and analytic approaches, permit a richer and more expansive understanding of people’s first-hand experiences, perceptions, and ways of coping with the pandemic. One recent mixed-methods study used both qualitative and quantitative methods to illuminate the pandemic as an unexpected opportunity for growth, finding that, among North Americans, individuals with high levels of self-transcendent wisdom described a greater ability to connect with friends, family, and community during times of physical distancing, and found that the interaction between disengaged coping (e.g., distraction) and self-transcendent wisdom was significantly associated with *increased subjective well-being* during this time (Kim et al., 2021). In another qualitative study based in the United Kingdom, approximately a third of adults reported *improvements* in their close relationships through themes of greater communication, togetherness, sharing responsibilities, and support networks, highlighting the early lockdown period of the pandemic as an opportunity for growth and relational flourishing (Vowels et al., 2021). These emerging findings speak to the need for mixed-methods research to more fully illuminate COVID-19-unique experiences that may not be captured by existing measures and quantitative approaches. Using a mixed-methods approach with a multinational sample and adopting a positive psychological lens, the present study sought to inductively examine the ways in which participants perceived interpersonal connection and relationship-building as a possible form of coping during the pandemic, and then to statistically test the associations among coping, appraisal, and well-being. In particular, our mixed-method approach expands on strictly quantitative approaches by permitting a richer understanding of individuals’ experiences during the COVID-19 lockdown period as articulated *in their own words.* Our approach also expands existing qualitative and mixed-method research by elucidating the ways in which individuals perceived interpersonal connection during this time and empirically demonstrating how such perceptions related to well-being.

While individual-level factors such as appraisal and coping may indeed influence people’s psychological responses during COVID-19, the broader sociocultural context also provides a backdrop in which certain kinds of psychosocial responses may be differently aligned with cultural norms and national public health policies. Careful consideration of cultural and country-level factors that may influence psychosocial responses is important in determining whether links between coping strategies and well-being during the pandemic vary across nations or are more universal. In the current study, we focused on the United States (U.S.), Japan, and Mexico in order to capture samples broadly representative of the distinct sociocultural contexts in North America, Asia, and Latin America, respectively.

Research has documented how culture influences individuals’ preferred coping strategies and socioemotional responses. Broadly speaking, individualistic cultures, such as those found throughout North America and Western Europe, tend to encourage primary control strategies that emphasize personal influence, agency, and self-expression consistent with an internal locus of control, whereas collectivistic cultures, such as those found in Asia and Latin America, tend to encourage secondary control strategies that promote adjustment and accommodation to the situation and more expressive restraint consistent with an external locus of control (Kim and Sherman, 2007; McCarty and Shrum, 2001; Weisz et al., 1984). Accordingly, people from individualistic cultures are more likely to use approach-focused and interpersonal coping strategies to influence their environment in the pursuit of personal goals, whereas people from collectivistic cultures are more likely to engage in more passive and avoidant coping (for a review, see Chun et al., 2006). For example, Mexican samples have shown evidence of more positive reframing and less self-distraction consistent with approach coping (Farley et al., 2005) as well as more collaborative and accommodative interpersonal coping (Gabrielidis et al., 1997) in comparison to Non-Hispanic U.S. Whites. And data also suggest that Asian and Asian American groups are less likely to use expressive disclosure and social support to solve problems (e.g., Taylor et al., 2004; Wang et al., 2010), but find implicit support (Benjamin et al., 2021; Taylor et al., 2007) and interdependent forms of mutual helping (e.g., Wang and Lau, 2015) to be more beneficial. To date, it is unclear whether these cultural preferences are reflected in people’s ways of coping during the pandemic and how coping and culture may interact to predict personal well-being outcomes. As such, the current study provides the first empirical exploration, to our knowledge, of country (U.S., Japan, or Mexico) as a moderator of the links between coping strategies and subjective well-being during COVID-19.

On a national level, it is critical to consider that the U.S., Japan, and Mexico also differed in their COVID-19 governmental and public health responses, which may have further contributed to variation in individuals’ well-being and coping strategy use. First, these countries varied in their degree of COVID-19 impact and exposure. According to Our World in Data (Ritchie et al., 2020), which relies on data from the WHO for confirmed cases and deaths, on July 29th, 2020 (the day our data collection began), the U.S., Japan, and Mexico had a cumulative total of 12,909.87, 257.37, and 3,656.50 confirmed cases of COVID-19 per million people, respectively, and 457.08, 8.08, and 488.39 COVID-19 deaths per million people, respectively. In addition, these three countries had varying degrees of social distancing measures in place. Generally speaking, across all three nations, the most severe lockdown measures instated in March of 2020 were lifted around the time of our study’s launch (July/Aug 2020). In Japan, the state of emergency was lifted by May 25, 2020, although strict international travel restrictions and high levels of societal compliance with COVID-19 protocols remained (Kusama et al., 2023). In Mexico, Mexico City was taken out of lockdown from mid-June 2020 onward and largely maintained a lenient pandemic policy with few social restrictions (Sheridan, 2021; Froese et al., 2021). In the U.S., COVID-19 responses quickly became politically polarized, with more politically conservative states (e.g., Texas) lifting stay-at-home orders as early as April 30th, 2020 (Linnane, 2020), while more politically liberal states (e.g., New Hampshire) maintained stay at home orders well into mid-June (Downey, 2021), with COVID-19 protocol compliance varying vastly within and between states. The present investigation thus captures a unique timeframe during which individuals across the world began transitioning out of the strictest social distancing measures and were gradually adjusting to the “new normal” of living with the pandemic under moderate restrictions.

### 1.1 The current study

The COVID-19 era is exemplified by global disruption and profound loss with subsequent research focused on the negative impacts of the pandemic on psychosocial functioning and well-being, with only a limited body of research addressing the role of positive coping strategies in mitigating the stress-distress link. Furthermore, studies have largely neglected testing these questions using multinational samples that have the potential to reveal variability or universality in these processes. Last, the COVID-19 research literature has been primarily quantitative in nature, using existing scales and constructs to approximate the unprecedented experience of living during COVID-19, and has not fully harnessed the power of mixed-method approaches that also incorporate qualitative analysis to more richly understand COVID-19-specific phenomena and impacts on well-being.

The current study is a mixed-method investigation of coping strategies and well-being among individuals from three countries in the wake of the initial COVID-19 lockdown period (i.e., Summer of 2020). We drew samples from the U.S., Mexico, and Japan due to a cultural rationale based on existing research; the U.S. exemplifying high-individualism and Japan and Mexico exemplifying different forms of high-collectivism (e.g., harmony collectivism and convivial collectivism; Campos and Kim, 2017). These countries were also selected due to observed differences in their COVID-19 responses (e.g., distinct social distancing measures at the time of data collection).

Qualitative and quantitative data were collected as part of two larger cross-sectional online survey studies on cross-cultural differences in perceptions of social processes and social technology use (Study 1) and personal well-being (Study 2) during COVID-19, which were preregistered on Open Science Foundation repositories. The present study is a separate secondary analysis of these pre-registered datasets.

Using qualitative analytic approaches, we examined the different ways in which participants perceived and described interpersonal connection and relationship building during a unique time period characterized by social restrictions and relational disruption. Specifically:

Qualitative Research Question: *How did individuals experience interpersonal connection during the initial COVID-19 lockdown period? (*i.e.*, Summer 2020)*.

We gathered qualitative data to explore the specific cognitive appraisal of the COVID-19 experience being one of connection and relationship enhancement. Based on these findings, we then developed a questionnaire for COVID-related Connection Appraisal, which was validated using a new sample. Using *quantitative* analytic approaches, we then tested the associations of Approach Coping Style and COVID-related Connection Appraisal with three well-being outcomes (loneliness, distress, and happiness). We proposed two hypotheses and two exploratory research questions:

*H*1: Approach Coping Style will be associated with more favorable well-being outcomes (lower distress and loneliness, greater happiness; significance criterion α = 0.05).

*H*2: COVID-related Connection Appraisal will be associated with more favorable well-being outcomes (lower distress and loneliness, greater happiness; significance criterion α = 0.05).

*EQ*1: Are the relationships between adaptive coping (approach coping style, COVID-related connection appraisal) and personal well-being moderated by country? (α = 0.05)

*EQ*2: Is COVID-related Connection Appraisal distinct from Approach Coping Style in its association with well-being outcomes? (α = 0.05)

## 2 Qualitative methods

### 2.1 Participants

Qualitative data were collected from 141 adults from the United States (U.S.; *n* = 50), Japan (*n* = 41), and Mexico (*n* = 50). All participants were required to be over the age of 18 and to self-identify as a resident and national of the U.S., Mexico, or Japan. The mean age of participants was 29.66 (*SD* = 9.93) years and 54% of participants identified as male, 44% as female, and 2% as ‘other.’ Participants represented a wide range of socioeconomic backgrounds; in terms of income, 17% reported “living comfortably,” 34% reported “doing okay,” 30% reported “just getting by,” and 11% reported “finding it difficult to get by” (Federal Reserve, 2020). On average, participants reported 2.66 people (*SD* = 1.53) living in their household.

### 2.2 Procedure

Informed consent was obtained from all individual participants included in this study. Procedures were approved by the Institutional Review Board at Haverford College. Qualitative data were collected using the online survey platform Qualtrics in July–August of 2020, approximately 4–5 months after the initial COVID-19 lockdown in the U.S., Japan, and Mexico. Participants were recruited using Prolific, a widely-used web-based labor market for human subjects research. The survey was administered in English, Spanish, and Japanese for U.S., Mexican, and Japanese samples, respectively. Survey questions and responses were translated using forward-translation and back-translation methods (Brislin, 1970) with two English-Spanish bilingual researchers (including author LB) and two English-Japanese bilingual researchers. Participants were told that this study aimed to understand people’s relationship experiences during the pandemic, particularly during the initial lockdown/stay-at-home order period. Participants were required to spend at least 4 min responding to the following prompt and were unable to advance until they had done so:

*In what ways have you felt connected to others during this time? “Others” may include close or more distant relationships, old and/or new relationships. Please reflect on your thoughts, feelings, and experiences during COVID-19.*

At the end of the survey, participants completed a brief demographic questionnaire. All participants were compensated $1.60 USD for their time, in the form of Prolific payment.

### 2.3 Qualitative data analysis

Qualitative analysis of participants’ responses was conducted by authors LB and SW, who are two trained mixed-methods researchers specializing in culture, coping, and health and well-being with experience conducting and publishing qualitative research. LB is a Spanish-fluent White female researcher and SW is an Asian female researcher; both are located in the United States. Several aspects of Braun and Clarke (2022) thematic analysis were employed to identify themes in participants’ responses. The first phase of our qualitative analysis involved an open coding of participants’ responses, based on inductive approaches, in which both researchers independently read participants’ responses in batches of 10 and identified emergent themes, with a specific focus on the different ways in which participants described connection during the COVID-19 lockdown period. The researchers met regularly to discuss and clarify emergent themes and repeatedly returned to the data to ensure that all interpretations and identified themes were true to the data and corroborated by other participants’ responses (Elo et al., 2014; Pyett, 2003). To ensure the robustness of our qualitative analysis, themes were validated through a process of triangulation and peer debriefing. Initially, both researchers independently coded the data and generated preliminary themes. They then compared their coding and discussed any discrepancies, refining the themes until a consensus was reached. This iterative process continued until both researchers were confident that the themes accurately reflected the data. Throughout the process, the coding team engaged in critical self-reflection about any personal biases that may affect their assessment of participants’ responses and identification of themes in order to uphold the integrity of the qualitative research process.

## 3 Qualitative results

On average, responses were 82 words in length (*M* = 82.05, *SD* = 39.05; range: 13–232). Responses spanned a wide range of experiences and perspectives on the pandemic, varying in depth and emotional intensity; participants described experiences of connection with family members, friends, acquaintances, and even strangers. Qualitative analysis of participants’ responses not only provided insight into diverse ways of connecting with close relationships during the pandemic but also revealed profound reflections on the state of human connection. Most notably, throughout participants’ responses, *a broader cognitive appraisal of connectedness that prompts adaptive interpersonal behaviors* during the pandemic emerged, wherein people considered their views of the changing world, their relationships with people in it, and their awareness of the state of human connection during the early stages of the pandemic. This unique COVID-related appraisal process transcends existing appraisal conceptualizations and forms of coping (e.g., COVID-related challenge appraisal, Chu et al., 2022; social support use during COVID-19, Szkody et al., 2021) to frame the pandemic as a time of interpersonal connection that entails a broader awareness of the pandemic’s impact on one’s own relationships and those of others in the world. This unique conceptualization of connection incorporates both appraisal and subsequent interpersonal behavior together as a dynamic form of coping. To better illustrate this point, we provide examples of participants’ responses, spanning four overarching themes identified in our qualitative analysis. We note that each theme was reflected in responses across all three countries.

### 3.1 Interconnectedness with humanity

In many responses, participants described a sense of global connectedness with humanity, viewing the pandemic as *a world-shared experience.* Participants reflected on how this perspective affected their treatment of others, the relative strength of their relationships with others (compared to before the lockdown period), and their subsequent perceptions of relationships with loved ones.

“I felt a sense of togetherness, as I felt that everyone was together. When you are confined, your social status does not matter, because everybody is going through the same thing. In fact, I felt more connected with people during lockdown.” (27-year-old Japanese man).

“In the face of the current situation you realize that we are all connected. Like something that happens on the other side of the world affects us here. Like the decisions that I make or that someone else makes, significantly impact the solution to a large-scale problem. In a social manner despite the distance, we have been connected to family and friends in a virtual manner, suddenly the relationships become more important and you give yourself moments to nourish those that matter most.” (33-year-old Mexican woman).

### 3.2 Stronger existing relationships

A number of participants indicated that the pandemic fueled a sense of connection that prompted participants to think more about their relationships and seek opportunities to strengthen existing relationships with loved ones. Participants described going out of their way to find digitally-mediated ways to communicate with friends and family members, resulting in more frequent connection and a heightened sense of social connection.

“I have felt more connected in some ways to people because of the pandemic. My family in particular have been making an effort every month to get together over Zoom to see each other. I also talk to some of my friends more often. This means quite a bit because through all of this, it means that people are thinking of each other and take the time for you. I find myself thinking much more about others and wondering how they are during this time.” (25-year-old U.S. Black/African American woman).

“I usually talk to my family via Skype/Zoom since I live abroad but this is the first time we had all joined the same call. It is possible that I am even more connected now than I was before the pandemic. My friends at home had more time to chat because they are not working. I was able to talk with people who I have not spoken to for a long time, out of concern for each other’s situations.” (29-year-old Japanese woman).

### 3.3 Cherishing most important relationships

Several participants described the pandemic as an opportunity for reflection on one’s most important social ties. Many described an increased sense of closeness to friends and family and described appreciating existing relationships or more clearly treasuring certain relationships more than before, that in turn prompted more social outreach and initiation.

“Staying at home gives you more time to reflect on your life and your relationships with others as well. Since I have been home, I have realized what is most important to me. I have spent a lot more time reaching out to distant relatives and friends that I have not spoken to. It has helped me build a better relationship with them. I think that these renewed relationships will last beyond the pandemic.” (42-year-old U.S. White, Non-Hispanic woman).

### 3.4 Expanding new relationships

Although the pandemic placed a number of limitations on individuals’ physical mobility—limiting people’s ability to socialize in person, for instance—several participants described the lockdown period as prompting a desire to pay attention and seek opportunities to reconnect with previous social ties and even forge new ones, thus building and expanding their relational networks.

“I’ve also had more people actually reach out and check on me which makes me feel more connected because this whole pandemic experience is helping people see that other people are important. The pandemic has given people the chance to pay more attention to the people around them and people who maybe aren’t so close by, but makes them want to reach out and stay connected. The pandemic has caused so many problems, but this has given people a chance to reconnect.” (34-year-old U.S. Non-Hispanic White woman).

“There is also the matter of new friends, many of whom I have met online during this time. Without the time and ability to spend almost all day online, it is unlikely I would have met these friends, who are from all over the country and the world. I am able to share my personal experiences with them through text and voice chat, and because we almost all have time to spend online, we have become much closer.” (21-year-old U.S. Non-Hispanic White woman).

Taken together, the qualitative results highlight COVID-related connection appraisal as an adaptive coping strategy that integrates cognitive appraisals with interpersonal behaviors, which have the potential to enhance resilience during this era. These findings align with Lazarus and Folkman (1984) transactional model of stress and coping, which emphasizes the role of cognitive appraisals in the coping process. At the same time, the emergent theme of COVID-related connection appraisal expands this model by highlighting the dynamic interplay between appraisals and interpersonal actions during a global crisis.

## 4 Questionnaire development

Our qualitative analysis provided a snapshot of individuals’ perceptions of human connection during the COVID-19 pandemic and the various dimensions of connection appraisal that emerged among U.S., Japanese, and Mexican individuals. Based on the qualitative data, we developed a COVID-related Connection Appraisal Questionnaire (see Appendix 1) to measure unique perceptions of human connection during this time, with the goal of quantitatively capturing this unique COVID-specific conceptualization of connection incorporating components of both appraisal and behavior together as a dynamic form of coping.

Questionnaire items derived from our qualitative data were administered in September 2020 with a *separate* sample of *N* = 302 participants from the U.S., Japan, and Mexico (described in the Quantitative Methods section below). Exploratory Factor Analysis (EFA) was then conducted via IBM SPSS Amos 26 using maximum likelihood extraction with promax oblique (i.e., correlated factors) rotation (Costello and Osborne, 2005). All 302 participants were included in the model simultaneously to achieve the minimum sample size generally recommended for EFA (Clark and Watson, 2019). EFA supported a nine-item, four-factor model of the questionnaire, identified as (1) stronger existing relationships, (2) cherishing most important relationships, (3) interconnectedness with humanity, and (4) expanding new relationships, which paralleled the overarching themes identified in our qualitative analysis (see Appendix 1 for final items). The cumulative variance explained by the final EFA solution was 69.18%, all primary factor loadings were greater than.50 (range 0.52–0.76) and were statistically significant (*p*’s < 0.001), and all cross-loadings fell below 0.30, indicating an acceptable model fit (Fabrigar et al., 1999; Hinkin, 1998). We note that as these factors were highly correlated, conceptually similar, and represented different dimensions of the same underlying construct of *Connection Appraisal*, we opted to combine the items from each derived factor into a single composite score, calculated as the mean of the scores of these items. This composite score demonstrated good internal reliability (Cronbach’s alpha = 0.85; range 0.76–0.86 per country) and strong convergent validity; mean connection appraisal scores were positively correlated with theoretically related factors including approach coping style and happiness and were negatively correlated with inversely-related factors including loneliness and psychological distress, *p*s < 0.05 (see Table 1).

**Table 1:** Bivariate correlations for main study variables and demographic characteristics.

	1	2	3	4	5	6	7
1. Loneliness							
2. Distress	0.53**						
3. Happiness	−0.65**	−0.54**					
4. COVID-related connection appraisal	−0.43**	−0.13*	0.49**				
5. Approach coping style	−0.37**	−0.12*	0.41**	0.59**			
6. Age	−0.04	−0.25**	−0.02	−0.26**	−0.20**		
7. Socioeconomic status^a^	−0.27**	−0.25**	0.34**	−0.15**	0.11*	−0.02	

*p < 0.05. **p < 0.01. ^a^Socioeconomic status is coded as: 1 = Finding it difficult to get by (financially); 2 = Just getting by (financially); 3 = Doing okay (financially); 4 = Living Comfortably (financially).

## 5 Quantitative methods

### 5.1 Participants

Quantitative data were collected from a *completely separate* sample of 302 adults (*M_age_* = 31.25, *SD* = 10.91, range: 18–69; 53.4% Male, 45.7% Female, 0.9% Other) from the U.S. (*n* = 95), Mexico (*n* = 102), and Japan (*n* = 105) following the qualitative study. Participants were required to be over the age of 18 and to self-identify as a resident and national of the U.S., Mexico, or Japan. Table 2 displays descriptive statistics, group difference tests, and post-hoc analyses for demographic and primary study variables. Japanese participants were significantly older than Americans and Mexicans. Mexicans were significantly younger than all other groups. Additionally, Mexicans reported lower levels of perceived socioeconomic status (SES) compared to all other groups (Federal Reserve, 2020).

**Table 2:** Descriptive statistics and group differences on study self-report variables.

	United States (US)	Japan (J)	Mexico (M)	Significance test	*p*-value	Effect size	*Post hoc*
	Mean (SD)	Mean (SD)	Mean (SD)				
Well-Being							
Loneliness^1^	24.13 (5.45)	24.64 (5.24)	24.58 (5.56)	*F*(2, 297) = 2.59	0.109	*η^2^_partial_ =* 0.009	
Distress^1^	24.99 (9.15)	22.18 (10.39)	25.20 (9.17)	*F*(2, 297) = 1.29	0.276	*η^2^_partial_ =* 0.009	
Happiness^1^	4.48 (1.52)	4.08 (1.49)	4.60 (1.29)	*F*(2, 297) = 10.18	<0.001***	*η^2^_partial_ =* 0.064	J < M; J < US; US<M
Coping							
Approach coping style^1^	32.11 (7.11)	32.10 (5.15)	34.61 (5.98)	*F*(2, 297) = 4.72	0.010**	*η^2^_partial_ =* 0.031	US<M
COVID-related connection appraisal^1^	3.00 (0.85)	2.37 (0.76)	3.07 (0.69)	*F*(2, 297) = 17.10	<0.001***	*η^2^_partial_ =* 0.103	J < US, J < M
Demographic features							
Age	28.76 (10.68)	39.18 (9.10)	25.40 (7.57)	*F*(2, 299) = 63.54	<0.001***	*η^2^ =* 0.298	M < US; M < J; US<J
Socioeconomic status (%)				*χ*^2^(6) = 32.13	<0.001***	*v =* 0.31	
1. Finding it difficult to get by	9.5	7.6	10.8				
2. Just getting by	29.5	48.6	56.9				US<J; US<M
3. Doing okay	44.2	25.7	32.4				M < US
4. Living comfortably	16.8	18.1	0				M < US; M < J

*p < 0.05; **p < 0.01; ***p < 0.001. United States (*n* = 95), Japanese (*n* = 105), Mexicans (*n* = 102). For chi-square tests, the effect size index used was Cramer’s V; in general, 0.10 indicates a small effect, 0.30 indicates a medium effect, and 0.50 indicates a large effect. For ANOVA and ANCOVA, η^2^ and η^2^_partial_ were used, respectively; in general, 0.01 indicates a small effect, 0.06 indicates a medium effect, and.14 indicates a large effect.

1 Covariates appearing in the model are evaluated at the following values: Age = 31.25, Socioeconomic status = 2.48.

An *a priori* power analysis using G*power (version 3.1) suggested that a minimum of 261 participants was sufficient to detect a small-to-medium main effect size of $f^{2}$ = 0.04, providing power = 0.80 and significance criterion of 0.05 for a multiple regression with four total predictors. Hence, the current study was well-powered to detect significant effects.

### 5.2 Procedure

Informed consent was obtained from all individual participants included in this study. Procedures were approved by the Institutional Review Board at Haverford College. Data were collected using the online survey platform Qualtrics. U.S. and Mexican participants were recruited using Prolific and Japanese participants were recruited using the Japanese web-based labor market Crowdworks. The survey was administered in English, Spanish, and Japanese. We used validated Spanish and Japanese translations of scales when possible (UCLA Loneliness Scale: Russell, 1996; Brief COPE: Carver, 1997). When translations were not available, we used forward-translation and back-translation methods (Brislin, 1970) with two English-Spanish and two English-Japanese bilingual research assistants.

Data were collected in September of 2020, approximately 6 months after the initial COVID-19 lockdown in the U.S., Japan, and Mexico. Participants completed a series of questionnaires regarding their coping strategies, appraisals of the pandemic, loneliness, distress, and happiness during the initial COVID-19 lockdown period. We included five attention check items (e.g., *I was born on February 30th. True or False?*) and two validity check items (*I verify that I am eligible according to the demographic criteria;* and *I verify that I paid attention and made a good faith effort in my study participation*) to assess participants’ attentiveness and due diligence when completing the study. Participants who failed these items (*n* = 24) or spent less than 10 min completing the 20-min survey (*n* = 10) were eliminated from the study, resulting in a final sample size of *N* = 302. ANOVA did not find significant group differences on attention and validity check attrition, *F*(2, 333) = 1.45, *p* = 0.176. Participants were compensated $2.40 USD for their time, in the form of Prolific payment (for US and Mexican participants) or Crowdworks payment (for Japanese participants).

### 5.3 Measures

#### 5.3.1 Loneliness

Loneliness was measured using the revised 10-item UCLA Loneliness Scale (Russell, 1996). Participants used a 4-point scale from 1 (*never*) to 4 (*always*) to respond to questions including “How often do you feel you lack companionship?” The UCLA Loneliness Scale has demonstrated good internal reliability and construct validity with various clinical and community samples (Russell, 1996). Cronbach’s alpha in the current sample was.83 (range 0.83–0.85 across groups).

#### 5.3.2 Psychological distress

Psychological distress was measured using the 10-item Kessler Psychological Distress Scale (K10) (Kessler et al., 2002), which asks participants to report the frequency of anxiety and depressive symptoms over the past 30 days using a 5-point scale from 1 (*none of the time*) to 5 (*all of the time*). A composite distress score was calculated by adding up participants’ scores on each item with higher composite scores indicating higher levels of distress. In clinical settings, a score of less than 20 indicates that the patient is “likely to be well”; 20–24 suggests “mild distress”; 25–29 suggests “moderate distress”; and greater than 30 suggests “severe distress.” The K10 has demonstrated good internal reliability and construct validity with clinical and community samples (Andrews and Slade, 2001). Cronbach’s alpha in the current sample was 0.94 (range 0.93–0.96 across groups).

#### 5.3.3 Happiness

Happiness was measured using the 4-item Subjective Happiness Scale (SHS) (Lyubomirsky and Lepper, 1999), which assesses an individual’s overall subjective happiness using a 7-point scale from 1 (*less happy*) to 7 (*more happy*). A composite happiness score was calculated by taking the mean of participants’ scores on each item with higher composite scores indicating higher levels of happiness. The SHS has demonstrated excellent internal reliability and construct validity across U.S. and diverse international samples (Lyubomirsky and Lepper, 1999). Cronbach’s alpha in the current sample was 0.91 (range 0.89–0.93 across groups).

#### 5.3.4 Approach coping style

Consistent with prior work, approach coping strategies (i.e., strategies aimed at dealing actively with the stressor or related emotions) during the pandemic were defined by and measured with six subscales from the 28-item Brief COPE (Carver, 1997): Active Coping, Use of Emotional Support, Use of Instrumental Support, Positive Reframing, Planning, and Acceptance. Prior research examining the factor structure of the Brief COPE scale has identified two major factors underlying the scale, identified as approach coping and avoidant coping (Eisenberg et al., 2012). The items of the six subscales mentioned above loaded onto the approach coping factor, did not cross-load onto the avoidant coping factor, and demonstrated good internal consistency in adults (Eisenberg et al., 2012). Measurement of approach coping using the items of these subscales has also been used in existing COVID-19 research on adults with excellent internal consistency (Shamblaw et al., 2021). In the current sample, participants responded on a 4-point scale from 1 (*I have not been doing this at all*) to 4 (*I’ve been doing this a lot*) to items including “I’ve been trying to see it in a different light, to make it seem more positive” (positive reframing) and mean approach coping composite scores were used. Cronbach’s alpha in the current sample was 0.84 (range 0.81–0.88 across groups).

#### 5.3.5 COVID-related connection appraisal

The newly-developed nine-item COVID Connection Appraisal Questionnaire, assessed the degree to which participants appraised the pandemic as a connecting experience. Participants responded on a 5-point scale from 1 (*Not at all*) to 5 (*Very much*) to items displayed in Appendix 1. A mean connection appraisal score was calculated as the arithmetic mean of all nine questionnaire items, with higher scores indicating greater connection appraisal. In the present sample, Cronbach’s alpha was 0.85 (range 0.76–0.86 across groups).

## 6 Quantitative data analysis

Quantitative data were analyzed using SPSS Version 28.0. Prior to running our primary analyses, we computed descriptive statistics (Table 2). We then computed group differences (by country) for each study variable using ANCOVA and Chi-Square statistics (Table 2). We then computed bivariate correlations between primary study variables and demographic variables (Table 1). Demographic variables were included as covariates in our models if they were significantly associated with both a dependent variable (loneliness, distress, or happiness) and an independent variable (approach coping style or COVID-related connection appraisal). Age and SES were subsequently included as covariates.

To test the relationship between coping strategies and well-being (Hypothesis 1 and 2), we ran a series of hierarchical linear regression analyses, controlling for age and SES, with coping style (approach coping style or COVID-related connection appraisal) predicting well-being outcomes (loneliness, distress, or happiness) (Tables 3, 4).

**Table 3:** Hierarchical linear regression of approach coping style on loneliness, distress, and happiness.

	Loneliness						Distress						Happiness					
Variable	B	SE	𝛽	*t*	*p*	∆R^2^	B	SE	𝛽	*t*	*p*	∆R^2^	B	SE	𝛽	*t*	*p*	∆R^2^
Step 1						0.072						0.127						0.117
Age	−0.02	0.03	−0.05	−0.88	0.38		−0.23	0.05	−0.26	−4.72	<0.001***		0.00	0.01	−0.01	−0.22	0.83	
SES	−1.75	0.37	−0.27	−4.77	<0.001***		−3.02	0.64	−0.26	−4.72	<0.001***		0.61	0.10	0.34	6.28	<0.001***	
Step 2						0.128						0.021						0.147
Age	−0.06	0.03	−0.12	−2.32	0.02*		−0.25	0.05	−0.28	−5.21	<0.001***		0.01	0.01	0.07	1.28	0.20	
SES	−1.49	0.34	−0.23	−4.32	<0.001***		−2.83	0.64	−0.24	−4.44	<0.001***		0.53	0.09	0.30	5.95	<0.001***	
Approach coping style	−0.32	0.05	−0.37	−6.90	<0.001***		−0.23	0.09	−0.15	−2.67	0.008**		0.09	0.01	0.38	7.48	<0.001***	

**Table 4:** Hierarchical linear regression of COVID-related connection appraisal on loneliness, distress, and happiness.

	Loneliness						Distress						Happiness					
Variable	B	SE	𝛽	*t*	*p*	∆*R*^2^	B	SE	𝛽	*t*	*p*	∆*R*^2^	B	SE	𝛽	*t*	*p*	∆*R*^2^
Step 1						0.066						0.127						0.117
Age	−0.02	0.03	−0.05	−0.88	0.38		−0.23	0.05	−0.26	−4.72	<0.001***		0.00	0.01	−0.01	−0.22	0.83	
SES	−1.75	0.37	−0.27	−4.77	<0.001***		−3.02	0.64	−0.26	−4.72	<0.001***		0.61	0.10	0.34	6.28	<0.001***	
Step 2						0.176						0.028						0.211
Age	−0.08	0.03	−0.16	−3.16	0.002**		−0.27	0.05	−0.30	−5.44	<0.001***		0.02	0.01	0.11	2.32	0.021*	
SES	−1.33	0.34	−0.20	−3.96	<0.001***		−2.71	0.64	−0.23	−4.26	<0.001***		0.48	0.09	0.27	5.63	<0.001***	
COVID-related connection appraisal	−2.88	0.34	−0.44	−8.40	<0.001***		−2.05	0.65	−0.18	−3.14	0.002**		0.844	0.09	0.48	9.69	<0.001***	

*p < 0.05; **p < 0.01; ***p < 0.001.

To determine whether country moderated these relationships (Exploratory Question 1), participants’ nationalities were coded into two dummy codes: Japanese and Mexican (coded as 1 s) with U.S. Americans coded as the referent group (0). We computed step-wise hierarchical linear regressions with covariates (age and SES) included in the first step, the independent variable (approach coping style or COVID-related connection appraisal) in the second step, the two dummy codes in the third step, and the centered interactions of the independent variable with each dummy code in the fourth step.

To assess whether COVID-related connection appraisal explained the relationship between approach coping style and well-being (Exploratory Question 2), we used Hayes’ PROCESS macro to compute a mediation model for each well-being variable, specifying approach coping style as the independent variable for each model and loneliness, distress, or happiness as the dependent variable, controlling for age and SES as covariates. To determine the statistical significance of the indirect effect of COVID-related connection appraisal on well-being outcomes, PROCESS uses percentile bootstrapping procedures (Preacher and Hayes, 2008). Unstandardized indirect effects were computed for each of 5,000 bootstrapped samples and the 95% confidence interval was computed by determining the direct effects at the 2.5th and 97.5th percentiles. If the 95% confidence interval did not include zero, the indirect effect was considered a significant mediator.

## 7 Quantitative results

Table 2 displays descriptive statistics, group difference tests, and post-hoc analyses for demographic and primary study variables. ANCOVA (controlling for age and SES) found significant group differences on happiness, approach coping style, and COVID-related connection appraisal. Japanese reported lower levels of happiness compared to all other groups; Mexicans reported higher levels of happiness compared to all other groups. Mexicans reported higher levels of approach coping style than Americans. Japanese reported lower levels of COVID-related connection appraisal compared to all other groups. ANCOVA did not find group differences on loneliness or distress.

Table 1 displays correlations between main study variables and demographic characteristics. Happiness and COVID-related connection appraisal and approach coping style were positively correlated, *p* < 0.01. Loneliness and distress were positively correlated, *p* < 0.01. Distress and loneliness were both negatively correlated with happiness, COVID-related connection appraisal, and approach coping style, *p*s < 0.05.

Tables 3, 4 displays hierarchical linear regression analyses, controlling for age and SES, with approach coping style and COVID-related connection appraisal predicting well-being outcomes (loneliness, distress, and happiness). Hierarchical linear regressions found significant negative main effects of approach coping style on loneliness and distress, and a positive main effect of approach coping style on happiness, *p*s < 0.01. Hierarchical linear regression analyses also found significant negative main effects of COVID-related connection appraisal on loneliness and distress, and a positive main effect of COVID-related connection appraisal on happiness*, p*s < 0.01. With regard to EQ1, country did not moderate any of these relationships, *p*s > 0.05.

**Figure 1:**
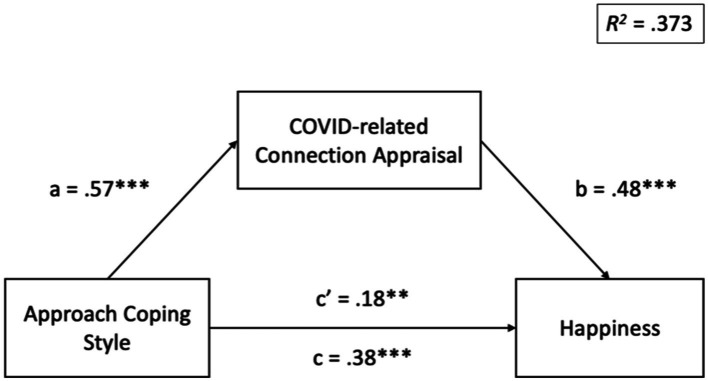
Standardized regression coefficients for the relationship between approach coping style and happiness as mediated by COVID-related connection appraisal. Indirect effect = 0.27, 95% CI: [0.14, 0.29]. **p < 0.01, ***p < 0.001.

**Figure 2:**
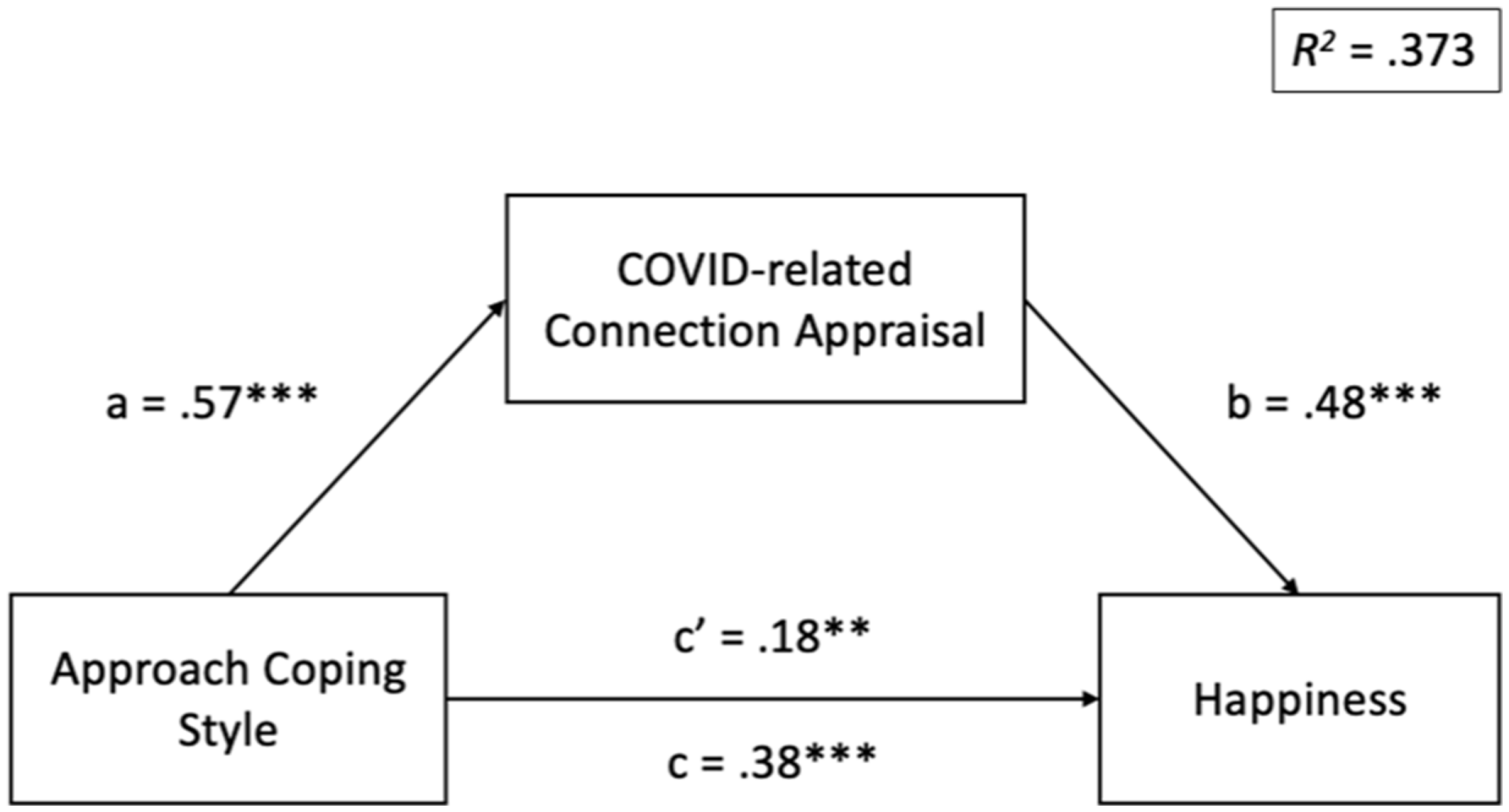
Standardized regression coefficients for the relationship between approach coping style and loneliness as mediated by COVID-related connection appraisal. Indirect effect = −0.25, 95% CI: [−0.27, −0.11]. **p < 0.01, ***p < 0.001.

With regard to EQ2, we conducted exploratory hierarchical linear regressions to examine whether the association between the general approach coping style was explained specifically by COVID-related connection appraisal. Results indicate that COVID-related connection appraisal significantly mediated the effect of approach coping style on happiness, 95% CI: [0.14, 0.29], as well as the effect of approach coping style on loneliness, 95% CI: [−0.27, −0.11]. COVID-related connection appraisal did not mediate the effect of approach coping style on distress, 95% CI: [−0.16, 0.001]. See Figures 1, 2 for standardized regression coefficients of each model.

## 8 Discussion

The current multinational, mixed-methods study investigated people’s experiences of connection and coping during the initial lockdown period of the pandemic characterized by social restrictions and relational disruption. Qualitative analyses illuminated the specific cognitive appraisal of the COVID-19 experience being one of interpersonal connection and relationship enhancement, that led to the development of a questionnaire to measure connection appraisal during this time. Quantitative analyses using this questionnaire with a second sample revealed positive associations among approach coping style, COVID-related connection appraisal, and all well-being outcomes (i.e., lower distress and loneliness, greater happiness). These associations did not differ by country, signaling potential universality in the links between coping processes and well-being. Finally, COVID-related connection appraisal mediated the relationship between approach coping and two well-being outcomes (loneliness and happiness), suggesting a distinct benefit of connection appraisal beyond the benefits of general trait approach coping style.

Across all three nations, qualitative analysis revealed profound reflections on the pandemic as an opportunity for expanded and strengthened personal relationships, with numerous participants describing the pandemic in terms of its unexpected silver linings (e.g., the stay-at-home order as an opportunity to strengthen connections with household members). While the preponderance of COVID research has understandably focused on the deleterious psychological consequences of the pandemic, our focus on an emergent COVID-related connection appraisal unearths an under-researched COVID-specific phenomenon wherein respondents described changed interpersonal perspectives (and subsequent behavior) on connection with humanity, stronger relationships, expanded new relationships, and a cherishing or revisiting of important relationships. This finding resonates with results from other mixed-methods studies, including that of Kim et al. (2021), who found that individuals with high levels of self-transcendent wisdom described a greater ability to connect with friends, family, and community during the lockdown period compared to before the pandemic and, accordingly, experienced increased well-being during this time. Our qualitative findings also align with those of Chu et al. (2022), whose quantitative study showed that emotion-focused coping combined with COVID-specific *challenge appraisals* was associated with psychological growth among U.S. college students, and those of Yeung et al. (2023), who found perceived social support to be associated with adversarial growth among Hong Kong nurses. Our results provide further qualitative evidence of people’s perceptions of the pandemic as an opportunity for positive relational transformation and highlight potential psychosocial benefits of perceiving the pandemic as an opportunity for growth, positive change, and increased connection. Our findings also expand existing literature by using *multi-national samples (in the U.S., Japan, and Mexico),* suggesting that these COVID-specific appraisals transcend a single cultural context.

As predicted, quantitative analyses found robust relationships between approach coping style and all three well-being outcomes (lower distress and loneliness, greater happiness). This finding aligns with existing pre-COVID research, which has traditionally regarded approach coping as a more adaptive and effective buffer of distress symptoms (versus avoidant coping; Eisenberg et al., 2012; Monzani et al., 2015). Our findings indicate that the general distress-buffering effects of approach coping style apply in the unique context of the COVID-19 pandemic and may be a potential pathway for resilience during this time. Interestingly, although group differences were found on approach coping style (U.S. < Mexico) and happiness (Japan < U.S. < Mexico), consistent with other cross-cultural studies of coping and subjective well-being (Chun et al., 2006), the relationships between approach coping style and well-being outcomes *did not differ by country*. This set of findings may suggest that while cultural factors indeed influence people’s preferences for different coping styles, approach coping, when actually used, holds the potential to improve well-being regardless of cultural context. Our findings offer preliminary evidence of potential universality in the effects of approach coping style on three forms of well-being in the context of the pandemic, although additional longitudinal research is needed to determine temporal ordering of such effects.

Using our newly developed questionnaire to assess COVID-related connection appraisal, we found that, consistent with our predictions and prior literature on growth-and supportive openness-related appraisals during COVID-19 (Diotaiuti et al., 2023), COVID-related connection appraisal was robustly related to all three well-being outcomes. Furthermore, our subsequent mediation analysis found COVID-related connection appraisal to be a significant mediator of approach coping style’s linkages with both loneliness and happiness, providing evidence that COVID-related connection appraisal is one specific pathway or mechanism by which general approach coping style is associated with well-being outcomes. This finding illustrates the additional explanatory power held by COVID-related connection appraisal as a unique form of adaptive coping that is distinct from existing approach coping strategies in its influence on well-being. We note that while our findings offer a fascinating snapshot of people’s cognitions and forms of coping during the COVID-19 era—highlighting a unique form of coping that incorporates both appraisal *and* related behavioral components—we are unable to ascertain whether the appraisal components of *Connection Appraisal* precede the behavioral components, as Folkman and Lazarus (1988) model would suggest, or vice versa. Perhaps cognition appraisal and behaviors were, in particular, integrated or indistinguishable during the unique context of a global pandemic in which normative social functioning was disrupted and coping processes were heightened. Relatedly, while our mediation analysis illuminates connection appraisal as a potential mechanism through which approach coping is associated with well-being during COVID-19, without repeated measures, we are unable to examine these constructs sequentially and cannot definitively say whether approach coping precedes connection appraisal or *vice-versa*. Rather, by examining these constructs concurrently, our cross-sectional findings highlight approach coping and connection appraisal as theoretically related but *distinct* forms of coping that differ in their associations with well-being outcomes. Moreover, harnessing a diverse multi-national and mixed-methods dataset, our findings underscore the significance of human connection as both an evaluative process and an active coping mechanism, highlighting the resilience and adaptability of individuals navigating unique time in our history. As Kim and colleagues articulate, *“If anyone is lucky enough to feel that they have grown as a result of the current pressures to self-isolate, it is likely people who have focused on the key things in life: forging connections and finding meaning”* (Kim et al., 2021, p. 3).

Our results speak to the benefit of mixed methodological approaches to investigate new phenomena. While the bulk of existing COVID-19 research has relied on deductive quantitative approaches to examine coping, our mixed-method investigation took an inductive-sequential (QUAL → QUAN) approach (Morse and Niehaus, 2009; Schoonenboom and Johnson, 2017), using qualitative analytic methods to explore new constructs (i.e., COVID-related connection appraisals) and then quantitatively testing pandemic-specific models of coping and well-being. Our *qualitative* analysis permitted us to capture people’s real-time appraisals of their social connections during a window of the pandemic characterized by social and physical distance, while our *quantitative* findings highlighted the universal effects of these appraisals, alongside traditional measures of coping, on corresponding well-being outcomes, permitting us to capture multiple dimensions of the coping process across culturally diverse nations. Taken together, these findings underscore the importance of promoting and facilitating social connections during crises and have a number of applications for public policy and clinical practice. Given the potential benefits of approach coping and connection appraisal to multiple facets of well-being during COVID-19, policymakers and mental health practitioners should consider strategies to mitigate social isolation and foster supportive community networks as part of emergency response plans. For instance, implementing virtual social programs or community support groups may help maintain social bonds and provide emotional support during times of physical distancing. These findings also offer potential clinical applications for cognitive behavioral therapy, in which challenge appraisals that frame solidarity and togetherness in the face of global stressors may be beneficial. Finally, our research highlights the need for culturally sensitive approaches to mental health interventions, recognizing that the ways people appraise and cope with crises may vary across different cultural contexts. Integrating these insights into policy and practice may enhance the resilience and well-being of diverse populations in the face of future global challenges.

### 8.1 Study limitations and future directions

The results of the current study should be considered in the context of the following limitations. First, with this cross-sectional correlational study, we cannot infer temporal ordering or causality between the variables; while we may rely on theory to infer causality, further research is required to explore causal links using a longitudinal design. In a similar vein, we recognize that the use of mediation analyses with cross-sectional data is potentially problematic, given their inability to detect temporal associations between variables; we note that these analyses were exploratory, and we proceed with caution when interpreting such findings. We also acknowledge limitations associated with the development of the COVID-related Connection Appraisal Questionnaire. Namely, all three countries were statistically treated as a single sample, as smaller sample sizes within groups (~100 participants per country) precluded us from conducting an individual EFA for each country, as is generally recommended by psychometricians (Clark and Watson, 2019). While our use of inductive-sequential (QUAL → QUAN) approaches to derive measure items was a relative strength of our study, a more rigorous examination of the resulting questionnaire’s factor structure using a separate EFA for each sample would be beneficial. We also acknowledge potential effects of response bias due to the online data collection mode; the current study may overrepresent individuals with reliable internet access and familiarity with online survey tools. Additionally, the demographic characteristics of our sample, such as the relatively younger age range and higher educational attainment, may limit the generalizability of our findings. Future research should aim to include more representative samples to enhance the external validity of findings. Another study limitation pertains to our data collection timeline. As data were deliberately collected during a specific window—approximately 6 months after the initial COVID-19 outbreak—our results may not generalize to other time periods throughout the pandemic. Further analysis examining these constructs during a different period of the pandemic or as an evolving longitudinal process in multinational samples is an important future direction.

## 9 Conclusion

Consistent with previous qualitative research that has shone a light onto positive psychological processes in response to COVID-19 (e.g., Kim et al., 2021; Vowels et al., 2021), this research contributes additional insights into how cognitive appraisals related to interpersonal connection can help individuals transcend challenges of the current moment above and beyond the general benefits of approach coping styles. Furthermore, cognitive appraisals about interpersonal connection should be considered separately from traditional conceptions of social support use that have been found to be protective in the stress process for COVID-19 (Szkody et al., 2021). Taken together, the findings of this mixed-methods, multinational study contribute to the growing understanding of how cognitive appraisals relevant to meaning-making, coping strategies, and well-being may intersect in the face of global suffering, highlighting approach coping style and connection appraisals as potential pathways for positive growth and psychological resilience in the face of unprecedented global transformation.

## Data Availability

The datasets presented in this study can be found in online repositories. The names of the repository/repositories and accession number(s) can be found at: https://osf.io/vrc4z?view_only=6f9ea4a616384449ad73a419a90286a4.

## Appendix 1

**COVID-related Connection Appraisal Questionnaire Items:**

*Stronger Existing Relationships:*

1. During this time, I often go out of my way to socialize with people using technology.
2. I communicate with my loved ones more frequently than I did before the pandemic.

*Cherishing Most Important Relationships:*

1. I feel a greater closeness to my friends and family.
2. This time has allowed me to reflect on and prioritize the social connections that I value the most.
3. I cherish my friendships and relationships more than I did before the pandemic.

*Interconnectedness with Humanity:*

1. I feel a greater sense of responsibility to help the people in my community.
2. I feel more connected to the world and all people.

*Expanding New Relationships:*

1. The pandemic has allowed me to meet new friends and acquaintances.
2. The pandemic has allowed me to reconnect with old friends and acquaintances.

Participants responded on a 5-point Likert scale from 1 (*Not at all*) to 5 (*Very much*) to each item displayed above. A mean connection appraisal score was calculated as the arithmetic mean of all nine questionnaire items, with higher scores indicating greater connection appraisal. Spanish and Japanese translations are available upon email request to the first author.
